# Promotion of breastfeeding in Italian Maternity Hospitals: a pre-intervention study

**DOI:** 10.1186/s13052-024-01793-9

**Published:** 2024-10-25

**Authors:** Riccardo Davanzo, Guglielmo Salvatori, Mariella Baldassarre, Irene Cetin, Elsa Viora, Elena Scarpato, on behalf of the “HPB Project” Working Group

**Affiliations:** 1 Nutrition Research Centre, University of Insubria, Varese, Italy; 2 Neonatal Intensive Care Unit, Bambino Gesù Children’s Hospital, IRCCS, Rome, Italy; 3 Department of Interdisciplinary Medicine-Neonatology and NICU, University “Aldo Moro”, Bari, Italy; 4 UOC Obstetrics, Fondazione IRCCS CA’ Granda, Hospital Maggiore Policlinico, Milan, Italy; 5 Department of Biomedical and Clinical Sciences, University of Milan, Milan, Italy; 6 President Elect, Italian Society of Obstetrics and Gynecology (SIGO), Turin, Italy; 7 Department of Translational Medical Sciences – Section of Pediatrics, University “Federico II”, Naples, Italy

**Keywords:** Breastfeeding, Postnatal practices, Hospital policy, Bundle intervention, Training, Infant feeding rate

## Abstract

**Background:**

In Italy, exclusive breastfeeding (EBF) rates at hospital discharge range unsatisfactorily between 20–97%.

**Methods:**

In 2023, the Project for Hospital Policy on Breastfeeding (HPB) has been launched to promote breastfeeding in Italian Maternity Hospitals (MHs) as a joint initiative of the Italian Scientific Societies involved in perinatal care together with the National Midwife (FNOPO) and Nurse (FNOPI) Boards and with Vivere Onlus, a family association. The HBP Project has been designed as an uncontrolled before-after study to increase EBF rate at hospital discharge in the population of healthy, term infant with a normal weight at birth following an intervention bundle comprising: 1) Establishment of a local hospital Working Group; 2) Adoption of a hospital policy; 3) Implementation of breastfeeding monitoring; 4) Training for perinatal care professionals; 5) Enhanced implementation of the practices of skin-to-skin contact (SSC) and mother-baby rooming-in; 6) Development/Improvement of perinatal care protocols.

**Results:**

We report the pre-intervention assessment of 89 out of the 111 enrolled MHs (80.2%) at the beginning of the Project (Time 1 or T1). Almost all MHs (96.6%) have a multi-professional Breastfeeding Working Group, while a hospital policy on breastfeeding is available only in 48.2%. Moreover, only 56.2% of the 9,777 perinatal health workers have been trained in breastfeeding. Over a 1-month period, SSC has been practiced in the delivery room by 76.9% of 6,304 term healthy newborn infants and rooming-in by 83.4% of 6,735 healthy term newborns of normal weight at birth. Over a 4-month period, 69.1% of 33,367 healthy term newborns of normal birth weight were exclusively breastfed at hospital discharge. Noticeably, EBF rate of MHs ranges from 4% up to 100%, the second quartile being 73%.

**Conclusion:**

At T1 of the HPB Project, breastfeeding rates at hospital discharge for healthy, term infants with a normal weight at birth appear to be suboptimal among Italians MHs. Particularly, the range of EBF rates among participating centers is wide, with 50% of the MHs having EBF rate lower than 73%. Therefore, the ongoing HPB Project might represent not only an opportunity to increase the initiation of breastfeeding and to improve quality of health care in the whole study group of MHs, but possibly also to level differences between centers.

**Supplementary Information:**

The online version contains supplementary material available at 10.1186/s13052-024-01793-9.

## Background

Mother’s milk represents the ideal food for the majority of newborns and infants, and breastfeeding is undoubtedly part of a healthy, both economically and ecologically advantageous lifestyle, not only for the mother-infant dyad but also for the family as a whole [1]. International [2] and national Health Authorities [3], and Scientific Societies [4] recommend early and EBF for the first 6 months of life, and to continue breastfeeding, together with complementary foods, up to the second year of life and beyond. Permanent monitoring of breastfeeding at Maternity Hospital (MH) discharge as well as along the first year of life represent essential population health indicators [5].

In Italy, monitoring of breastfeeding rates, particularly at hospital discharge, is currently performed only in few Regions, although data from a national survey of the Ministry of Health (MoH) show a wide variation of EBF rates among hospitals, ranging from 97% down to only 20% [6]. These data in addition to more recent studies [7] suggest the need for interventions to increase breastfeeding initiation rates.

In fact, among the multiple cultural, social, economic, educational, psychological and clinical determinants of breastfeeding. Health Services, health professionals and particularly pediatricians [8]. play a pivotal role in promotion, protection and support of breastfeeding [9]. Effective promotion of breastfeeding in MHs requires the implementation of appropriate postnatal practices such as SSC, early initiation of breastfeeding, rooming-in of the mother and the newborn, and limitation of formula milk supplementation to breastfed infants. These practices are part of the Steps proposed by the Baby Friendly Hospital Initiative (BFHI) [10–12], that, unfortunately, during the past 30 years has been proceeding slowly, at least in Italy, achieving at a national level a goal of less than 1% baby friendly MHs [13].

A limited commitment of health professionals in the promotion of breastfeeding, possibly due to a poor cultural investment and/or a poor recognition of the value of breastfeeding, has been identified as one of the reasons for persisting suboptimal postnatal practices and for the lack of competent support to breastfeeding [14]. Consequently, training on breastfeeding of health professionals has been identified as a relevant issue.

With these premises, the Project for Hospital Policy on Breastfeeding (HPB; in Italian: Progetto PAA) has been launched in early 2023 to promote breastfeeding in Italian MHs, independently of the BFHI, as a joint initiative of the Italian Scientific Societies involved in perinatal care (the Italian Society of Neonatology-SIN, the Italian Society of Pediatrics-SIP, the Italian Society of Pediatric Nutrition-SINUPE, the Italian Society of Obstetricians and Gynecologists-SIGO, the Italian Association of Hospital Obstetricians & Gynecologists-AOGOI, the Italian Society of Neonatal Nurses-SIN-INF and the Italian Society of Pediatric Nurses-SIP-INF), together with the National Midwife (FNOPO) and Nurse Boards (FNOPI) and with Vivere Onlus, a family association.

The HPB Project has the primary objective to assess the efficacy of a bundle intervention to increase the EBF rate at hospital discharge in a cohort of MHs.

Here, we illustrate the design of the study and provide baseline data collected at the beginning of the HPB Project (T1), that will allow comparison with data that will be collected at the end of the HPB Project (T2).

## Methods

Italy has around 68.5 million inhabitants and 411 MHs. In 2023, just 379,000 babies were born. The mean maternal age at childbirth in Italy has gradually increased, and it is now 32.5 years of age (https://www.istat.it/it/files//2024/03/Indicatori_demografici.pdf). Although breastfeeding rates, particularly at discharge from MHs, are not well documented, the available data show unsatisfactory results. The need for the promotion of breastfeeding is addressed by the Italian Law, but till now nationwide initiatives for MHs are essentially limited to the BFHI, as the Task Force on Breastfeeding of the MoH lacks a strong mandate to allow incisive intervention in this field.

In this context, in early 2023, the national multi-professional steering committee of the HPB Project invited all the Directors of the Departments of Obstetrics & Gynecology, and the Departments of Pediatrics and Neonatology of the 411 Italian MHs, to voluntarily take part to the HPB Project. The full commitment to the HPB Project was also confirmed by the Director of the Health Authorities and/or of the MHs.

**Fig. 1:**
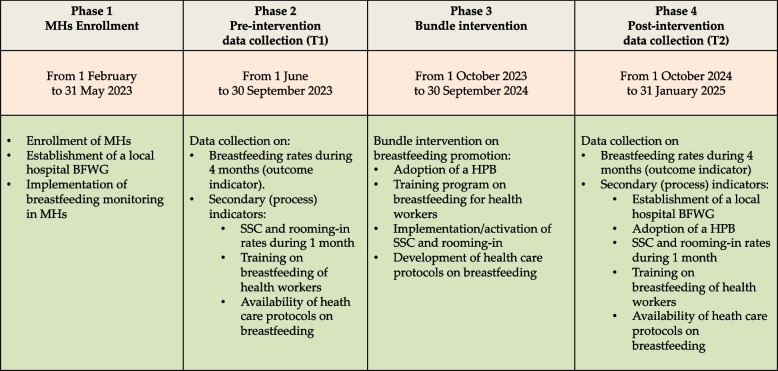
Schematic representation of the HPB Project study design. The HPB Project comprises 4 Phases with 2 data collection periods: T1 at the beginning and T2 at the end of the Project. MHs: Maternity Hospitals; BFWG: Working Group on Breastfeeding; HPB: Hospital Policy on Breastfeeding; SSC: Skin-to-Skin Contact

Between February and May 2023, 62 Italian Health Authorities including 111 MHs were enrolled in a 2 years Project (Fig. 1). This sample is composed of public MHs except one, although all are included in the National Health System. These MHs, being smaller local hospitals as well as university and tertiary care centers provide different level of care to a number of childbirths ranging from around 500 up to more than 3,000 per year, per single center.

The HPB Project has been designed as an uncontrolled before-after study to assess the efficacy of an intervention bundle to increase the EBF rate, to foster the development of a hospital policy on breastfeeding and to implement/reinforce SSC at birth and mother-baby rooming-in. The bundle intervention comprises the following items:

1. Establishment of a local hospital Working Group on breastfeeding, including at least the professions of pediatrician/neonatologist, nurse, obstetrician/gynecologist and midwife, and possibly that of anesthesiologist and the figure of family representative.
2. Adoption of a hospital policy on breastfeeding, not necessarily bound to full compliance with the International Code of Marketing of the Breast Milk Substitutes, thus bearing the collaboration with infant food companies regulated by the Italian Law [15].
3. Avoidance of formula milk prescription at hospital discharge to breastfed newborn infants, when breastfeeding mothers are self-effective.
4. Monitoring of breastfeeding rates at hospital discharge using the 1991 WHO definitions on infant feeding [16]. Healthy, term (≥ 37 weeks gestational age; GA) newborns, with normal weight at birth (≥ 2500 g), that stayed in the rooming-in ward continuously from childbirth until hospital discharge represents the population selected for infant feeding monitoring. Neonates with transient admission to Neonatology Department/Neonatal Intensive Care Unit (NICU) or neonates transferred from rooming-in to other wards or hospitals were excluded. Data on mother’s milk feeding and on donor human milk feeding were recorded separately, while both ultimately contributed to the calculation of the EBF rate.
5. Activation/reinforcement of an on-site and/or online training program on breastfeeding for perinatal care workers. The on-site training option was represented by the BFHI course for maternity staff on breastfeeding and by the online training resources specifically developed between 2021 and 2023 by SIGO/AOGOI for obstetricians/gynecologists (8 h course) and by SIN, SIP, SIN-INF, SIP-INF for pediatricians/neonatologists (12 h course), residents in Pediatrics (11 ½ hours course), nurses (11 h).
6. Implementation/reinforcement of two main postnatal practices known to facilitate breastfeeding: skin-to-skin contact (SSC) at birth and mother-neonate rooming-in.
  - SSC was defined as holding the baby naked against mother’s skin during the first 2 h after birth. The effective implementation of SSC has been documented by the check list in use for the prevention of postnatal collapse, in a selected population of healthy, term neonates [17, 18], during a 1-month period (between September 15th and October 15th 2023 at T1).
  - Rooming-in was intended as an extensive rooming-in of at least 20/24 h, accepting a 4 h maximum separation between mother and neonate, that comprises visits, procedures or intentional delivery of the neonate to the Nursery staff by the mother. The effective implementation of rooming-in was evaluated by a questionnaire submitted to the mothers at hospital discharge in a selected population of healthy, term neonates with normal weight at birth, during a 1-month period (between September 15th and October 15th 2023 at T1).
7. Development of 20 perinatal/postnatal care protocols on breastfeeding.

A frequency analysis on data collected at T1 and quartiles calculation for breastfeeding rates at hospital discharge was performed by a Digital Specialist of iDea Group. A more complex statistical analysis will be performed after T2 data collection.

## Results

### Hospitals enrolment

**Fig. 2:**
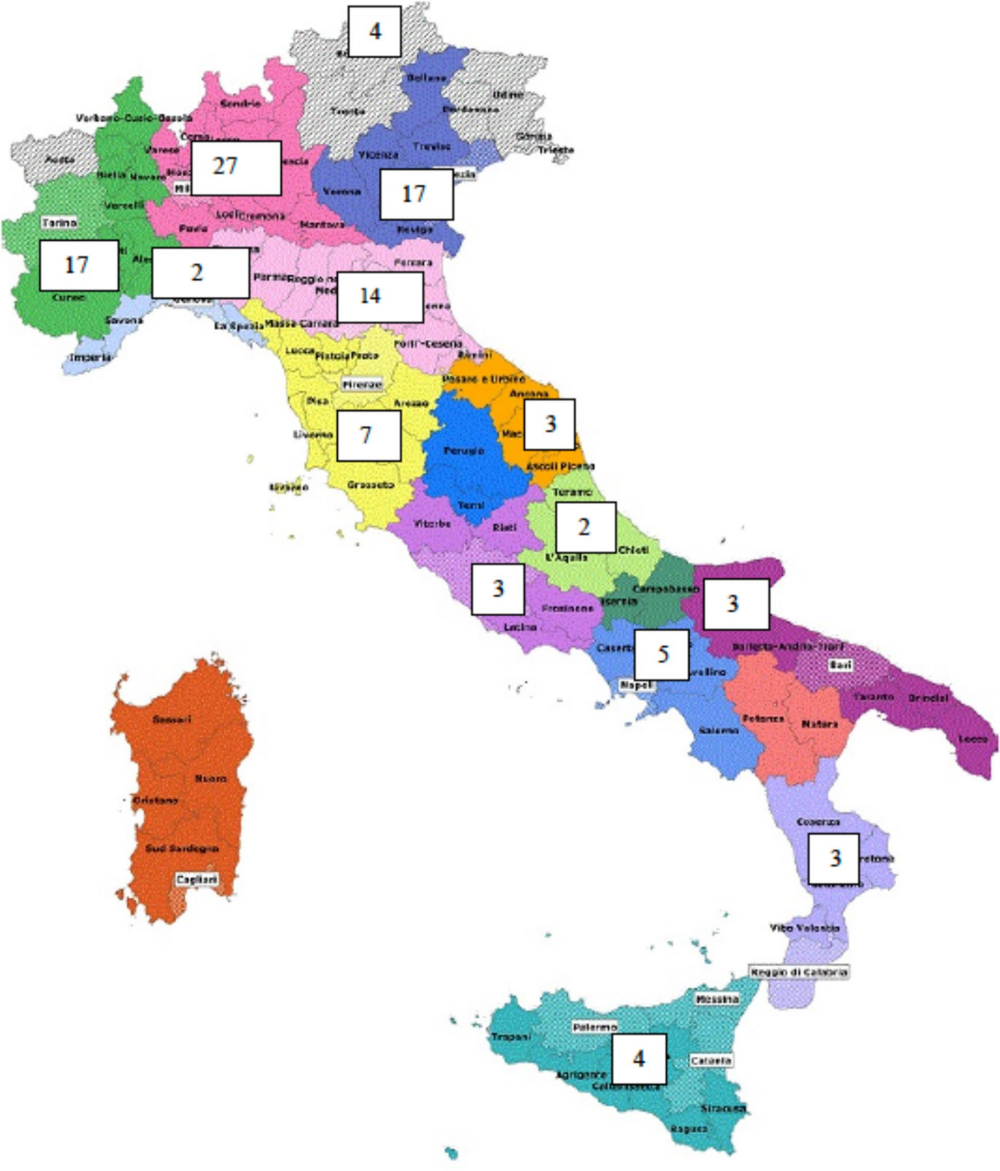
Geographical distribution of the 111 Maternity Hospitals participating to the HPB Project

The HPB Project enrolled 111 MHs in 14 out of 21 Italian Regions and Autonomous Provinces, that account for 27.0% of the Italian facilities, with a markedly higher prevalence of facilities from Northern Italy compared to Central and Southern Italy (Fig. 2). One hundred three out of 111 (92,8%) MHs completed the T1 online Survey. Data of the present report refers to the 89 centers (80.2%) that transmitted data before November 30th, 2023.

### Hospital breastfeeding working group

Almost all MHs (86/89; 96.6%) reported to have a multi-professional breastfeeding Working Group, although a family representative was included in only 35.9% (31/89).

### Hospital policy on breastfeeding

A hospital policy on breastfeeding is available in 43 out of 89 hospitals (48.2%). The contents of these 43 breastfeeding policies have been revised according to the check list shown in Table 1. Noticeably, only few policies are adequately communicated to mothers/families. Moreover, the indication to mothers/families of volunteer consultants in breastfeeding (e.g.: La Leche League) is considered only by a minority of hospital policies.

**Table 1:** Topics covered by the policies on breastfeeding in 43 Italian Maternity Hospitals

*Topics covered by the breastfeeding policies*	*Hospital breastfeeding policies covering the topic*	*Percentage*
1. Approval by Hospital/Health Authority director	27/43	62.8%
2. Communication to all staff	21/33	63.6%
3. Communication to mothers/families a. via website b. via poster c. via brochure	9/33 7/33 2/33	27.2% 21.2% 6.1%
4. Institution of a Maternity Hospital/Health Authority breastfeeding working group	32/42	76.2%
5. Overt statement that hospital staff is committed to promote and support breastfeeding	42/43	97.7%
6. Revision of the contents of the antenatal classes in order to give pregnant women appropriate information on breastfeeding	37/42	88.1%
7. Respect of the informed choice of a mother not to breastfeed	42/43	97.7%
8. Implementation of SSC, rooming-in and responsive breastfeeding	42/43	97.7%
9. Involvement of the breastfeeding working group in case of future changes of postnatal practices possibly affecting breastfeeding	32/43	74.4%
10. Information to mothers on the available community resources to support breastfeeding	42/43	97.7%
11. Information to mothers on the availability of volunteer consultants to support breastfeeding in the community (e.g. La Leche League)	19/43	44.2%
12. Avoidance of formula milk prescription to self-effective breastfeeding mothers	41/43	95.4%
13. Need for training on breastfeeding of the hospital staff	37/42	88.1%
14. The need for breastfeeding monitoring at hospital discharge	34/42	80.9%

### Postnatal practices

- SSC at childbirth is practiced in 85 out of 89 (95.5%) centers, although in 43.8% only after a vaginal delivery (Table 2). In the 80 MHs where SSC after vaginal delivery is recorded, 4,846 out of 6,304 (76.9%) healthy term newborns infants experienced SSC in the delivery room.
- According to the T1 Survey, 100% of the centers (89/89) state to practice rooming-in of at least 20/24 h. However, in the 81 MHs where this practice is recorded, rooming-in was experienced only by 5,615 out of 6,735 (83.4%) healthy term newborns with a normal birth weight.

**Table 2:** Implementation of skin-to-skin contact between mothers and healthy, term newborn infants in 89 Italian Maternity Hospitals

Practice of SSC	Maternity Hospitals (N)	Percentage
• Only after vaginal delivery	39	43.8%
• After vaginal delivery as well as CS	46	51.7%
• SSC not practiced	4	4.5%
Total	89	100%

*SSC* Skin-to-skin contact, *CS* Caesarean section

### Breastfeeding rates

In a 4-month monitoring of infant feeding, 69.1% (range. 4–100%; second quartile-Q2: 73%) healthy term newborns with normal weight were exclusively breastfed at discharge (Table 3). A small percentage of neonates (1.8%) was also given donor human milk. Moreover, the low rate of predominant breastfeeding (2.1%) shows that the use of glucose solution as a pre lacteal feed is limited. Finally, only 4.7% of the study sample did not receive any human milk from birth to hospital discharge.

**Table 3:** Breastfeeding rates at hospital discharge in 89 Italian Maternity Hospitals among healthy term newborns with normal birth weight

	Neonates (N)	Percentage
Exclusive breastfeeding	23,054	69.1%
o Only mother’s milk	22,447	67.3%
o Mother’s milk and/or donor human milk	607	1.8%
Predominant breastfeeding	704	2.1%
Complementary feeding	8,033	24.1%
No breastfeeding	1,576	4.7%
Total	33,367	100%

### Training on breastfeeding

The combined percentage of health workers involved in perinatal care and trained on breastfeeding is 56.2%, with a wide variation from 23.4% of the health and social care workers from the Obstetrics & Gynecology Department up to 70.2% of the pediatric nurses (Table 4). In general, the coverage for the training on breastfeeding is higher for healthcare professionals from the Pediatric and/or Neonatology Department. Moreover, training on breastfeeding of health and social care workers shows a lower coverage when compared with that of physicians, midwives and nurses of the same department.

**Table 4:** Training on breastfeeding of health workers in 89 Italian Maternity Hospitals

Health workers per site of work	Trained/total health workers (N)	Percentage
*Obstetrics & Gynecology Department*		
• Obstetricians/Gynecologists	421/1,289	32.7%
• Midwives	1,914/2,816	67.9%
• Nurses	261/560	46.6%
• Health and social care workers	218/904	24.1%
Subtotal	2,814/5,569	50.5%
*Pediatric Department/Nursery*		
• Pediatrician	269/458	58.7%
• Nurses	626/892	70.2%
• Health and social care workers	45/192	23.4%
Subtotal	940/1,542	61.0%
*Neonatology Department/Nursery*		
• Neonatologists	435/623	69.8%
• Nurses	1,211/1,786	67.8%
• Health and social care workers	94/257	36.6%
Subtotal	1,740/2,666	65.3%
Total (all staff)	5,494/9,777	56,2%

### Breastfeeding protocols

Most participating hospitals adopted only a limited number of health care protocols among those identified as relevant to the promotion and support of breastfeeding by the HPB Project Working Group (Table 5). In particular, protocols on prevention of hypoglycemia, SSC after vaginal delivery and breastfeeding topics for antenatal classes were adopted by ≥ 80% of the MHs. On the contrary, protocols on the prevention and management of lactational mastitis, thermal control of the newborn, management of early neonatal weight loss and support of breastfeeding in jaundiced neonates were the most neglected.

**Table 5:** Protocols on breastfeeding promotion and support in 89 Italian Maternity Hospitals; protocols are listed in decreasing order of availability

	Hospitals that adopted the protocol (N)	Percentage
1. Prevention of neonatal hypoglycemia	84	94.4%
2. SSC after vaginal delivery	82	92.1%
3. Check list on breastfeeding topics for antenatal classes	73	82.0%
4. Prevention of sudden unexpected postnatal collapse (SUPC)	69	77.5%
5. Zero separation and rooming-in 24/24 h	67	75.3%
6. Storage of expressed mother’s milk and human donor milk	66	74.2%
7. Responsive breastfeeding	58	65.2%
8. Prevention of in hospital neonatal fall	57	64.0%
9. Helping mothers to breastfeed	57	64.0%
10. Hospital discharge of the breastfed newborn	56	62.9%
11. Prevention and management of pain while breastfeeding	53	59.5%
12. Expression of breast milk	52	58.4%
13. Supplementing breast milk with formula	51	57.3%
14. SSC after CS	51	57.3%
15. Contraindications to breastfeeding	50	56.2%
16. Prevention and management of breast engorgement	50	56.2%
17. Prevention and management of lactational mastitis	48	53.9%
18. Thermal control of the newborn infant	47	52.8%
19. Management of early neonatal weight loss	47	52.8%
20. How to support breastfeeding in the jaundiced newborn infant during phototherapy	44	49.4%

*SSC* Skin-to-skin contact, *CS* Caesarean section

## Discussion

The present study presents the breastfeeding scenario in a sample of more than a quarter of Italian MHs. This study is aimed to assess the pre-intervention need to improve breastfeeding outcome at hospital discharge in MHs voluntarily included in a nationwide Project inspired to, but independent from, the BFHI. This thoughtful choice implies to renounce to the guarantees of an external tutorship as well as to the on-site assessment by UNICEF staff and to be indeed satisfied even with a partial adherence to the Code. At the same time, this choice has the strategic value to allow the enrollment of many MHs and to promote breastfeeding at the same time, possibly under the impulse given by the competition between centers.

Data presented in this paper refers to the T1 phase preceding a bundle intervention aimed to promote the start of breastfeeding.

### The breastfeeding policy

Our data show that although almost all hospitals (96.6%) report a breastfeeding working group, a hospital policy on breastfeeding is available only in 48.2% and, when available, it is suboptimal particularly in terms of communication of the policy to mothers/families and valorization of volunteer consultants to support breastfeeding after hospital discharge. In Italy, the scarce diffusion of hospital policies on breastfeeding has already been shown by a survey on MHs with NICU [19], while the present study involves MHs both with and without a NICU.

Unfortunately, the lack of a hospital evidence-based policy, routinely communicated to staff and parents, may jeopardize the start of breastfeeding, as a policy may provide an organizational and clinical standard set for health workers and ensure mothers receive consistent, evidence-based care [20, 21].

According to the BFHI, hospital policy on breastfeeding is an essential component of Step 1 (Step1b), the other two being full adherence to the International Code of Marketing of Breast-Milk Substitutes (the “Code”; Step 1a) and monitoring of breastfeeding (Step 1c) [12, 22, 23].

Participation to the HPB Project implies the development of a breastfeeding policy and the monitoring of breastfeeding, while only a partial adherence to the “Code” is required although in accordance with the Italian Law [15]. Specifically, the HPB Project “only” requires pediatricians/neonatologists not to prescribe formula milk at hospital discharge to breastfed neonates. Although this issue might be considered as a downsizing of the contents of the BFHI, by which the HPB Project is definitely inspired, it should be rather intended as a pragmatic way to achieve a wider and more rapid consensus on the goal of promoting breastfeeding in Italian MHs. After all, the policies adopted by the MHs may be updated in the future in terms of desirable higher level of breastfeeding protection.

### Postnatal practices

SSC between mother and baby, started as soon as possible after childbirth, is known to carry many benefits: better thermal control and relax for the baby, improved maternal-infant bonding and facilitation of both initiation and duration of breastfeeding. Consequently, it is recommended to start SSC immediately after birth, and to continue it uninterruptedly for at least 1 h, or until the first breastfeeding [24]. SSC at childbirth is reported to be practiced in most centers, although many fewer after a CS, possibly due to maternal health conditions, the need for a higher organizational complexity and for integrated care. Conversely, mothers and babies after a CS represent a subgroup that would indeed benefit most from an intimate and immediate SSC [25]. Around three quarters of healthy, term neonates are recorded to experience SSC during the first 2 h of life, although we have no information on the actual duration of the practice, that might actually reveal a fleeting and less valuable experience.

Rooming-in is recommended by UNICEF, World Health Organization [10], Italian Ministry of Health [26] and Italian Obstetric and Pediatric Scientific Societies [27, 28]. In fact, studies clearly show that, besides other benefits, rooming-in between mother and baby provides increased confidence in handling and caring for the baby, lower level of stress and opportunity for a responsive and more successful breastfeeding [29, 30]. Babies should not be separated unless in case of valid medical reasons or to ensure mother and/or baby safety. Obviously, health care staff should visit and supervise the dyad, providing help, when needed. We must recognize that rooming-in can represent a demanding experience for some women, especially when the physical and/or psychological conditions are not optimal [31, 32]. Thus, sometimes new mothers may reveal emotional distress and/or overt mental disorders, which should be promptly identified, providing support and timely treatment [33, 34]. In these cases, the healthcare team should assess the applicability and continuity of rooming-in in an individualized manner. In our study, rooming-in is practiced in all MHs of our sample, although medical records show that an extensive (20/24 h), if not round-a-clock rooming in, is experienced by only 4/5 neonates, a suboptimal figure, as the study population is made up of healthy term newborn infants of normal weight. Although we did not explore the reasons of mother-baby separation, nevertheless, referring to available published data and clinical practice, we can understand why: a) perinatal care practices is not sufficiently enabling mother to be ready and responsive to babies during rooming-in; b) mothers may have the expectation that the care of the neonate should be delegated to staff; c) health professionals may have a suboptimal competency to implement and maintain rooming-in; d) postpartum care for the dyads may show insufficient integration among different professionals; e) caregivers may have limited access to rooming-in, disregarding that partners and other family members may provide an essential practical and emotional support.

### Breastfeeding outcome at hospital discharge

The naturality of breastfeeding is not sufficient to guarantee an early and exclusive breastfeeding. In fact, many influencing factors like maternal inexperience, anxiety and lack of information, persisting infant feeding myths, not evidence based perinatal practices, improper attitude of health workers and last, but not least, an aggressive marketing of breast milk substitutes [35] explain why activation and functioning of a *warm chain* of support for breastfeeding is permanently required [36]. In many hospitals worldwide the use of formula supplementation in the first days after birth is still too casual, despite it is widely recognized that early and higher volume of formula supplementation negatively affect breastfeeding rate at hospital discharge [37] and that actually only a minority of newborn infants should require a medically justifiable supplementation [38, 39]. As a consequence, much more neonates than needed go home after birth being formula fed. On the contrary, neonates who are exclusively breastfed at hospital discharge not only are less likely to stop breastfeeding prematurely, but also carry a stronger potential of health. We collected information on the feeding status at hospital discharge of a national sample of more than 33,000 neonates, that accounts for nearly 8% of births in Italy [40]. The EBF rate in our study population seems to be satisfactory (almost 70%), although we should appreciate that it refers to a selected population of healthy, term neonates with a normal weight at birth, for which we would expect a better breastfeeding outcome at hospital discharge. Actually, the EBF rate of the whole study sample is the result of the contributions of 89 different MHs, characterized by very different EBF rates ranging from 4% up to 100%. Moreover, we should observe that around 50% of the MHs has indeed an EBF rate lower than 73% (Q2: 73%). This subgroup of MHs certainly witnesses higher needs and deserves stronger efforts. A previous study explored the initiation of breastfeeding in Italy [41], actually reporting higher EBF rate at a national level. Nevertheless, this study has been conducted on a smaller sample and was possibly prone to a recall bias, as the information regarding the categorization of the newborn feeding status was collected through interviews to mothers many days after birth.

### Training of healthcare personnel in breastfeeding

Perinatal health professionals have a duty to advocate for improving child health [42]. This duty includes promoting breastfeeding, regardless of their personal experience with infant feeding [43, 44] or whether their day-to-day activities include direct involvement with breastfeeding women and infants [45]. Health professionals are required to have a positive attitude toward breastfeeding, to be competent in understanding the importance of breastfeeding and the physiology of lactation, to manage common breastfeeding problems and to support or refer breastfeeding women for appropriate treatment [46]. Unfortunately, breastfeeding is insufficiently integrated in the pre-service education system and therefore corrective measures should be taken to provide in-service training.

The present study shows that, at least in Italy, training on breastfeeding is inadequate for all the basic perinatal professions (pediatricians/neonatologists, nurses, obstetricians and gynecologists, midwives), and above all for health and social care workers. As a positive reaction to the Government recommendation on specific education in breastfeeding for health professionals [46], in the last 2 years SIN, SIP, SIN-INF and SIPINF have produced specific online courses on breastfeeding for pediatricians/neonatologist, residents in Pediatrics and pediatric nurses. An online course on breastfeeding for midwives is going to be completed during 2024 also by the Midwifery Board of Italy (FNOPO). Finally, SIGO has offered a popular online course on breast diseases during lactation.

Although the use of online resources for training in breastfeeding may raise understandable doubts, an online approach to in-service education has been shown to be effective at improving practices and attitudes among health care professionals [47, 48]. In general, online training is more cost-effective than an equivalent in-person course and results in superior knowledge acquisition. On the contrary, e-learning tends to generate lower satisfaction scores when compared to instructor-led training. In fact, face-to-face training has specific advantages such as social interaction during training sessions, chance to get immediate answers to questions, more flexibility and personalization.

Actually, the HPB Project has recommended the use of both modes of training in breastfeeding for health professionals mainly due to two reasons. First, the significant progressive hospital staff shortage in Italy, with increasing difficulty to allow participation of health workers to in-person training courses also for few consecutive days. Second, the need to scale up training for health professionals rapidly within the deadline set by the project.

### Healthcare protocols related to breastfeeding

Breastfeeding is possibly hindered by some hospital activities, so that it deserves to be protected by clinical protocols that might integrate the promotion of breastfeeding with an appropriate evidence-based care for mother and infants.

A hospital protocol is an agreed standardized way of performing a task. Particularly, breastfeeding protocols state the course of action that should be adopted by health professionals regarding facilities organization and clinical practices. Obviously, protocols should be regularly reviewed and updated in the light of current research in order to ensure the best delivery of care. Besides the development of breastfeeding protocols by every single hospital, the Academy of Breastfeeding Medicine produces appreciated clinical protocols for the management of common medical problems, that may impact breastfeeding success (https://www.bfmed.org/).

Our study shows that breastfeeding protocols are inconstantly adopted by Italian MHs, thus possibly making health care related to breastfeeding less consistent. The present study demonstrates that a joint Initiative from different scientific societies involved in the area of perinatal care has succeeded to enroll as much as one quarter of Italian MHs, without any Government support. We believe that voluntary adhesion of so many MHs in the absence of any research fund allocated to peripheral centers is impressive. We presume that the participating centers have valued the goal of promoting the initiation of breastfeeding, have appreciated the work and the guidance of a national multi-professional Working Group, and have taken up the challenge of a quality improvement bundle intervention.

This study has also some limitations. First, health facilities participating to the HPB Project represent a self-selected group with stronger motivation to promote breastfeeding. Therefore, the true picture of breastfeeding in Italian MHs might be even less encouraging, as the centers with lower EBF rates at discharge probably did not join the HPB Project. Second, in the MHs’ sample, facilities from Northern Italy are more represented than those from Central and Southern Italy (73% vs 27%). Thus, the study sample might be more representative of a specific area of the country. Third, information on some relevant variables that characterizes a MH (such as the number of births per year and the CS rate) and might influence the efficacy of the bundle intervention to promote breastfeeding are not yet available, as they will be collected later in the course of the project at T2.

## Conclusions

The present study depicts a suboptimal breastfeeding scenario in Italian MHs as documented by unsatisfactory EBF rate for healthy neonates, deficiency of breastfeeding policies and protocols, scarce breastfeeding training of perinatal health professionals, and limited implementation of postnatal practices, such as SSC and rooming-in.

The ongoing HPB Project promoted by Italian Scientific Societies involved in perinatal care, the National Boards of Nurses and Midwives, and by Vivere Onlus might represent an opportunity not only for attaining better breastfeeding outcomes and to improve quality of care in the whole study group of MHs, but also to level differences between centers.

## Supplementary Information

Supplementary Material 1.

## Data Availability

The datasets used during the current study are available from the corresponding author on reasonable request.

## Abbreviations

AOGOI: The Italian Association of Hospital Obstetricians & Gynecologists
BFHI: Baby-Friendly Hospital Initiative
COM.A.SIN: Breastfeeding Section of the Italian Society of Neonatology
COVID-19: COronaVIrus Disease 19
EBF: Exclusive Breastfeeding
FNOPI: National Nurse Board
FNOPO: National Midwife Board
HPB: Hospital Policy on Breastfeeding
MH: Maternity Hospital
MoH: Ministry of Health
NICU: Neonatal Intensive Care Unit
PAA: Politica Aziendale sull’Allattamento
SIGO: Italian Society of Obstetricians and Gynecologists
SIN: Italian Society of Neonatology
SIN-INF: Italian Society of Neonatal Nurses
SINUPE: Italian Society of Pediatric Nutrition
SIP: Italian Society of Pediatrics
SIP-INF: Italian Society of Pediatrics Nurses
SSC: Skin-to-Skin Contact
SUPC: Sudden Unexpected Postnatal Collapse
TAS: Task Force on Breastfeeding
TASIP: Breastfeeding Section of the Italian Society of Pediatrics
UNICEF: United Nations International Children's Emergency Fund
WHO: World Health Organization
