# Supplementary material for: Promotion of breastfeeding in Italian Maternity Hospitals: a pre-intervention study
Source: Ital J Pediatr. 2024 Oct 25;50:219. doi: 10.1186/s13052-024-01793-9 (PMC11520110; doi:10.1186/s13052-024-01793-9)
Supplement: Supplementary file 1 — Supplementary Material 1. [file 13052_2024_1793_MOESM1_ESM.docx]

***FORM A (T1) for data collection at the beginning of HPB Project (Progetto PAA)***

Each Maternity Hospital (MH) should provide its own form.

| **Part A. Information on the MH** |
| --- |
| Heath Authority: |
| MH identification number |
| MH name: |
| Leader of the breastfeeding working group BFWG (responsible of data transmission):  1. Name 2. Surname 3. Mobile number: |
| 1. Is your MH a baby friendly hospital? |
| 1. Yes |
| 1. No |
| 1. No; nevertheless, the MH in on the BFH pathway |

| **Part B. BFWG** | | |
| --- | --- | --- |
|  | Yes | No |
| 1. Does a working group on breastfeeding (BFWG) exist in your MH? |  |  |
| 1. Is it truly multidisciplinary, including at least a pediatrician/neonatologist, an obstetrician, a midwife and a nurse? |  |  |
| 1. Does it include an anesthesiologist? |  |  |
| 1. Does it include a family representative? |  |  |

| **Part C. Hospital policy on breastfeeding** | **Yes** | **No** |
| --- | --- | --- |
| 1. Approval by Hospital/Health Authority director |  |  |
| 1. Communication to all staff |  |  |
| 1. Communication to mothers/families    1. via website    2. via poster    3. via brochure |  |  |
| 1. Institution of a Maternity Hospital/Health Authority breastfeeding working group |  |  |
| 1. Overt statement that hospital staff is committed to promote and support breastfeeding |  |  |
| 1. Revision of the contents of the antenatal classes in order to give pregnant women appropriate information on breastfeeding |  |  |
| 1. Respect of the informed choice of a mother not to breastfeed |  |  |
| 1. Implementation of SSC, rooming-in and responsive breastfeeding |  |  |
| 1. Involvement of the breastfeeding working group in case of future changes of postnatal practices possibly affecting breastfeeding |  |  |
| 1. Information to mothers on the available community resources to support breastfeeding |  |  |
| 1. Information to mothers on the availability of volunteer consultants to support breastfeeding in the community (e.g La Leche League) |  |  |
| 1. Avoidance of formula milk prescription to self-effective breastfeeding mothers |  |  |
| 1. Need for training on breastfeeding of the hospital staff |  |  |
| 1. The need for breastfeeding monitoring at hospital discharge |  |  |

| **Part D. Postnatal practices facilitating breastfeeding**  Tick the correct box | |
| --- | --- |
| Is SSC during at least 2 hours practiced with newborn infants **≥**37 GA? | |
| 1. No |  |
| 1. Yes, only after vaginal delivery |  |
| 1. Yes, also after CS |  |
| Does the MH use check list by the Italian Society of Neonatology on the prevention of SUPC prevention? |  |
| 1. Yes |  |
| 1. Yes, although just beginning the implementation |  |
| 1. No |  |
| Which is the number of SSC after vaginal delivery among healthy newborn infants **≥** 37 weeks GA during one-month period? |  |
| a) Number of SSC during one month |  |
| b) Total number of healthy newborn infants **≥** 37 weeks GA discharged during one month |  |
| Is extensive rooming-in (rooming-in >20 hours/24) practiced among healthy term newborn infants with a birth weight ≥ 2500 g? | |
| 1. Yes |  |
| 1. No |  |
| Which is the number of dyads who experience extensive rooming-in among healthy newborn infants **≥** 37 weeks GA with BW ≥ 2500 g in one-month period? |  |
| a) Number of extensive rooming-in during one-month period |  |
| b) Total number of healthy newborn infants **≥** 37 weeks GA with BW ≥ 2500 g discharged during one month |  |

| **Part E. Breastfeeding rate at hospital discharge** (according to Infant feeding definition by WHO, 1991). Data refer to healthy newborn infants **≥** 37 weeks GA and with a BW ≥ 2500 g during the period from 1 June until 30 September 2023. | |
| --- | --- |
|  | Number of neonates |
| - Mother’own milk |  |
| - Donor milk |  |
| - PBF |  |
| - Complementary feeding |  |
| - Formula feeding |  |
| - Total |  |

| **Part F. Training on breastfeeding of health workers** | |
| --- | --- |
| Health workers per site of work | Trained/total health workers  (N) |
| *Obstetrics & Gynecology Department* |  |
| - Obstetricians/Gynecologists |  |
| - Midwives |  |
| - Nurses |  |
| - Health and social care workers |  |
| Subtotal |  |
| *Pediatric Department/Nursery* |  |
| - Pediatrician |  |
| - Nurses |  |
| - Health and social care workers |  |
| Subtotal |  |
| *Neonatology Department/Nursery* |  |
| - Neonatologists |  |
| - Nurses |  |
| - Health and social care workers |  |
| Subtotal |  |
| Total (all staff) |  |

| **Part G. Protocols on breastfeeding** | | |
| --- | --- | --- |
|  | Yes | No |
| Does the MH have a check list on breastfeeding topics for antenatal classes |  |  |
| Does the MH have the following protocols? |  |  |
| 1. Thermal control of the newborn infant |  |  |
| 1. Prevention of in hospital neonatal fall |  |  |
| 1. Prevention of sudden unexpected postnatal collapse (SUPC) |  |  |
| 1. SSC after vaginal delivery |  |  |
| 1. SSC after CS |  |  |
| 1. Zero separation and rooming-in 24/24 h |  |  |
| 1. Contraindications to breastfeeding |  |  |
| 1. Responsive exclusive breastfeeding |  |  |
| 1. Helping mothers to breastfeed |  |  |
| 1. Prevention and management of pain while breastfeeding |  |  |
| 1. Management of early neonatal weight loss |  |  |
| 1. Supplementing breast milk with formula |  |  |
| 1. Prevention of neonatal hypoglycemia |  |  |
| 1. How to support breastfeeding in the jaundiced newborn infant during phototherapy |  |  |
| 1. Prevention and management of breast engorgement |  |  |
| 1. Expression of breast milk |  |  |
| 1. Storage of expressed mother’s milk and human donor milk |  |  |
| 1. Prevention and management of lactational mastitis |  |  |
| 1. Hospital discharge of the breastfed newborn |  |  |

**Annex 1. Questionnaire on rooming-in submitted to mothers at hospital discharge**

|  | Yes | No |
| --- | --- | --- |
| 1. Did your baby stay in your room all the desired time, even 24/24 hour? |  |  |
| 1. Did your baby stay with you all the previous nights? |  |  |
| 1. If your baby was separated from you, did the separation time exceeded 4 hours? |  |  |
|  | Yes | No |
| If the answered was “yes” to all the 3 above listed questions ,the dyad can be considered to have practiced extensive rooming-in |  |  |
